# An *invitro* dataset on infectious potential of transmitted/founder (TF) and non-TF (NT) HIV-1 viruses generated from Interferon alpha-treated primary CD4^+^ T cells

**DOI:** 10.1016/j.dib.2020.105365

**Published:** 2020-02-29

**Authors:** Manickam Ashokkumar, Aanand Sonawane, Balakumaran Sathiyamani, Bennett Henzeler Esakialraj L, Luke Elizabeth Hanna

**Affiliations:** a Department of HIV/AIDS, National Institute for Research in Tuberculosis, Chennai, India; b University of Madras, Chennai, India

**Keywords:** Human immunodeficiency virus, Mother-to-Child transmission, Transmitted founder viruses, Interferon alpha resistance

## Abstract

This data article describes the infectivity of transmitted/founder (TF) and non-TF (NT) HIV-1 viruses derived from primary CD4^+^ T cells treated with or without IFN-α, over a period of 12 days. TF and NT viruses described in this article were derived from the same individual (one of each from 8 infants who acquired HIV infection through mother-to-child transmission (MTCT). IFN-α resistance to both TF and NT viruses was studied by infecting TZM-bl cells and measuring luciferase expression (expressed as relative light units, RLU). Measurement of luciferase expression is extremely sensitive and allows quantification of even small changes in gene expression at the transcriptional level.

**Table:** Specifications Table

Subject	Virology
Specific subject area	HIV/AIDS
Type of data	Figure Supplementary table
How data were acquired	Luciferase expression (RLU), Infinite M200Pro plate reader (Tecan, Switzerland).
Data format	Raw (Supplementary table) Analyzed (Figure)
Parameters for data collection	Viruses derived from primary CD4^+^ T cells (treated with and without IFN-α); infection of TZM-bl cells with chimeric TF and NT viruses to investigate IFN-α resistance.
Description of data collection	Infectious potential of TF and NT chimeric viruses produced from CD4^+^ T cell cultures treated with or without IFN-α for varying periods of time was determined by quantification of infectivity in TZM-bl cells.
Data source location	Institution: National Institute for Research in Tuberculosis City/Town/Region: Chennai Country: India
Data accessibility	With the article

**Table:** 

**Value of the Data** • The data reveals a unique feature of TF viruses that distinguishes them from NT viruses. • The data provides a clue on one of the mechanisms that favour early HIV infection. • The protocol for studying IFN-α resistance may be useful for HIV researchers.

## 1 Data description

**Fig. 1:**
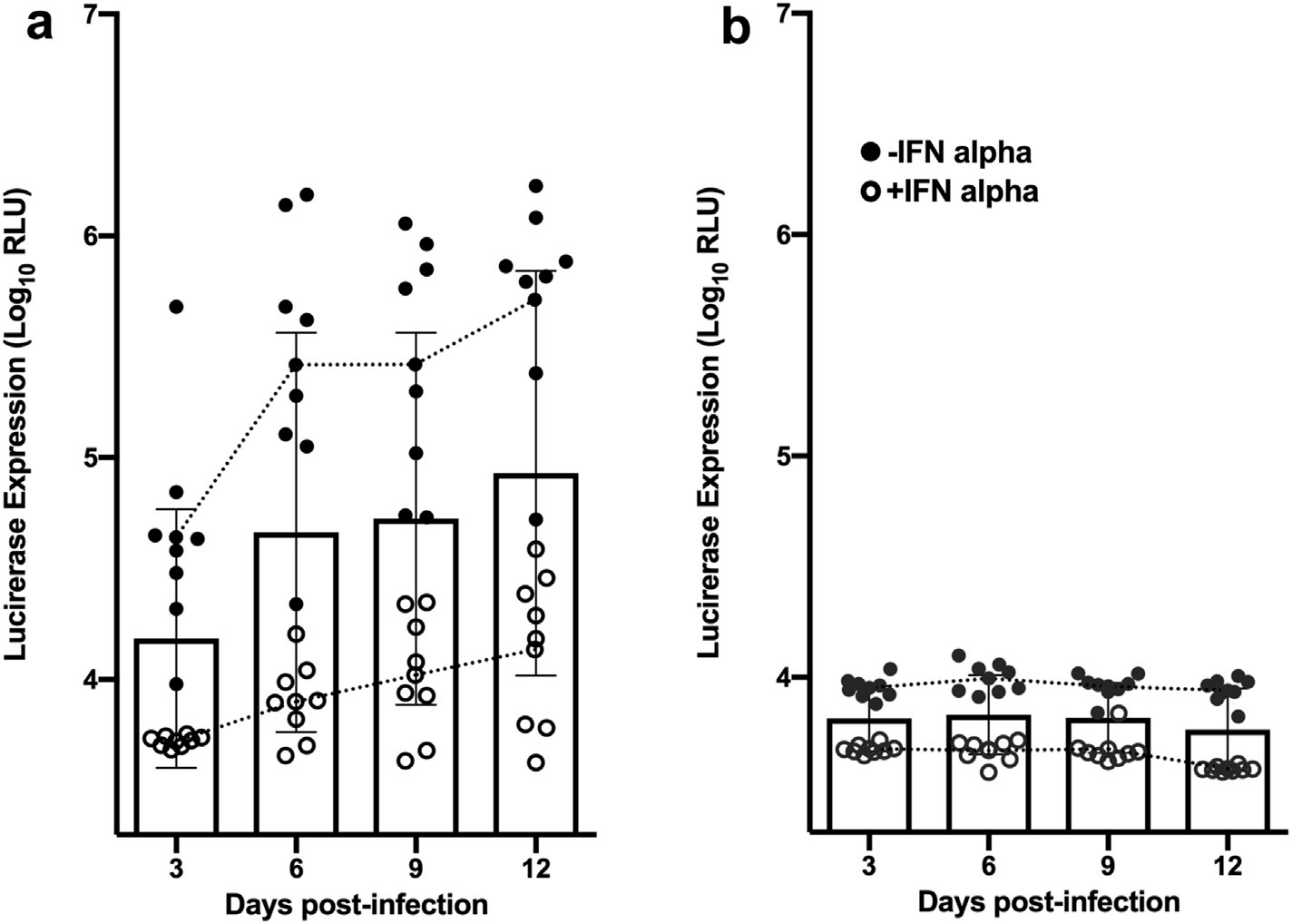
Infectivity of TF (a) and NT (b) viruses in TZM-bl cells. Infectious potential of chimeric TF and NT viruses derived from CD4^+^ T cells pre-treated with or without IFN-α at different time points was determined by measuring luciferase expression in TZM-bl cells after single round infection.

In this report, we present data on the infectious potential of TF and NT viruses generated from IFN-α treated primary CD4^+^ T cell cultures at different time points (3, 6, 9 and 12 days post infection). Resistance to IFN-α is believed to be an important property of HIV-1 isolates that establish new infection in the human host [1]. Infectivity of TF and NT viruses was determined by infecting TZM-bl cells and measuring luciferase expression in terms of relative light units (RLU). The normalized RLU data obtained after deducting the background (cell control) value is presented in Supplementary Table 1. TF viruses derived from both IFN-α treated and untreated CD4^+^ T cells were found to be more infectious than NT viruses derived from CD4^+^ T cell cultures grown under the same conditions (Fig. 1). Infectivity of TF viruses that were unexposed to IFN-α was found to be significantly higher than that of TF viruses grown from CD4^+^ T cell cultures exposed to IFN-α at all time points. On the other hand, the infectivity of NT viruses produced from IFN-α treated as well as untreated CD4^+^ T cells was not significantly different at any given time point. These findings indicate that TF viruses naturally possess enhanced resistance to IFN-α than NT viruses. The infectivity of TF and NT viruses exposed to IFN-α was similar at the earliest time point (day 3 post-infection), while that of TF viruses increased gradually with time (6, 9 and 12 days post-infection). The difference was statistically significant with p values of 0.015, 0.017 and 0.014 respectively as compared to NT variants.

## 2 Experimental design, materials, and methods

### 2.1 Cells

Primary CD4^+^ T cells were isolated from healthy donor peripheral blood mononuclear cells (PBMC) by negative selection using EasySep™ Human CD4^+^ T Cell Isolation Kit following the manufacturer's protocol. In brief, 5 × 10^7^ PBMCs/mL were mixed with the antibody coktail in PBS at a concentration of 50μl/mL. Non-CD4^+^ T cells labeled with antibodies and magnetic particles were separated using an EasySep™ magnet. The isolated CD4^+^ T cells were then stimulated with phytohemagglutinin (20 μg/mL) in the presence of 20 U/mL recombinant interleukin-2 (IL-2) in Rosewell Park Memorial Institute (RPMI) 1640 medium containing 10% (v/v) heat inactivated fetal bovine serum (FBS) (Sigma-Aldrich) at a concentration of 1 × 10^6^ cells per milliliter. TZM-bl indicator cell line (Lot number 150078) was maintained in Dulbecco's Modified Eagle medium (DMEM) with 10% FBS and 1% penicillin-streptomycin. Cells/cell lines were cultured at 37 °C with 5% CO_2_.

### 2.2 Virus production

Chimeric TF and NT viruses were derived from infants who acquired infection through MTCT as described previously [2]. The viruses were used to infect activated donor CD4^+^ T cells cultured in the presence or absence of IFN-α. Cell-free virus supernatants were collected at different time points (3, 6, 9- and 12-days post infection) and stored at −80 °C until use.

### 2.3 Luciferase expression

Virus supernatants collected at different time points were used to infect TZM-bl cells and viral infectivity was measured by quantifying luciferase expression using a Bright-Glo luciferase assay system (Promega) following the manufacturer's protocol. Briefly, TZM-bl cells were plated in a 96-well format (1 × 10^4^ cells/well) 24 hours before infection. At 48 hours post-infection, cells were lysed with 1X passive lysis buffer, and luciferase expression was measured in a luminometer and expressed as RLU. Data generated from the experiments were converted to logarithmic (log_10_) value before analysis. GraphPad Prism version 8 software was used for analysis and making of figure.
