# Supplementary material for: An invitro dataset on infectious potential of transmitted/founder (TF) and non-TF (NT) HIV-1 viruses generated from Interferon alpha-treated primary CD4+ T cells
Source: Data Brief. 2020 Feb 29;29:105365. doi: 10.1016/j.dib.2020.105365 (PMC7075791; doi:10.1016/j.dib.2020.105365)
Supplement: Multimedia component 1 [file mmc1.docx]

**Supplementary Table 1.** Luciferase expression (mean RLU) of TZM-bl cells infected with TF and NT viruses generated from IFN-α treated or untreated CD4+ T cells at different time points.

| **Sample ID** | **Cell control** | **3rd day** | | **6th day** | | **9th day** | | **12th day** | |
| --- | --- | --- | --- | --- | --- | --- | --- | --- | --- |
| **IFN alpha** |  | **−** | **＋** | **−** | **＋** | **−** | **＋** | **−** | **＋** |
| **IN03-TF** | 3367 | 44681 | 5464 | 1534700 | 16003 | 918170 | 22280 | 1206000 | 38702 |
| **IN04-TF** | 3023 | 20809 | 5425 | 189820 | 6625 | 198770 | 8469 | 655430 | 6278 |
| **IN05-TF** | 3058 | 43115 | 5673 | 417870 | 11007 | 706210 | 8715 | 729290 | 15258 |
| **IN06-TF** | 3398 | 38085 | 5552 | 478370 | 7861 | 578360 | 17224 | 767330 | 28736 |
| **IN07-TF** | 3637 | 478360 | 5016 | 1378900 | 9745 | 1137100 | 21879 | 1681300 | 19433 |
| **IN08-TF** | 3277 | 30210 | 5314 | 127470 | 8048 | 55041 | 11997 | 619960 | 24343 |
| **IN09-TF** | 3268 | 9532 | 4833 | 112340 | 5033 | 104590 | 4299 | 240160 | 6053 |
| **IN10-TF** | 3058 | 69997 | 5062 | 21879 | 4537 | 53796 | 4788 | 52660 | 4220 |
| **IN03-NT** | 3393 | 7591 | 4925 | 8165 | 5196 | 6919 | 4175 | 6648 | 4068 |
| **IN04-NT** | 3047 | 8365 | 4635 | 10943 | 5060 | 8604 | 4436 | 7993 | 3820 |
| **IN05-NT** | 3462 | 8236 | 4776 | 11408 | 4720 | 8825 | 4307 | 10123 | 3739 |
| **IN06-NT** | 3270 | 8796 | 5211 | 8933 | 5026 | 9238 | 6886 | 9594 | 3845 |
| **IN07-NT** | 3029 | 9623 | 4603 | 8610 | 4955 | 10431 | 4791 | 9546 | 3804 |
| **IN08-NT** | 3029 | 9203 | 4620 | 10554 | 3721 | 9340 | 4519 | 9199 | 3779 |
| **IN09-NT** | 3367 | 9334 | 4432 | 8710 | 4263 | 9479 | 4564 | 8441 | 3949 |
| **IN10-NT** | 3023 | 10905 | 4726 | 12571 | 4443 | 10388 | 4611 | 8603 | 3847 |
